# An energy efficient processor array and memory controller for accurate processing of convolutional neural network-based inference engines

**DOI:** 10.1038/s41598-025-23303-5

**Published:** 2025-11-12

**Authors:** S. Deepika, V. Arunachalam

**Affiliations:** Department of Micro and Nanoelectronics School of Electronics Engineering, Vellore Institute of Technology, Vellore, Tamil Nadu 632014 India

**Keywords:** Convolutional neural network, Dataflow, Deep learning, Energy-efficient accelerator, Image classification, Compression, Sparsity, Engineering, Mathematics and computing

## Abstract

Exploiting unstructured sparsity in the hardware accelerator of a Convolutional Neural Networks (CNNs) based inference can improve energy efficiency. However, it needs a complex controller for indexing and load-balancing. A controller for managing unstructured sparsity in Fully Connected (FC) layers is designed. In a pre-trained Visual Geometry-Group-16 (VGG-16) model, a ~ 20% sparsity is introduced using an induced sparsity mechanism. ImageNet dataset-based analysis of this model provides 95% classification accuracy and 0.96 harmonic mean of precision and recall. Each Input Feature Map (IFM) and its corresponding weight vector of an FC layer are arranged in a row of memory. A Combined IFM & Weights - Zero Valued Compression (CIW-ZVC) controller permits only the valid data from off-chip to on-chip memory. This is improving the data-movement rate with minimum hardware overhead. A processor array of 256 Convolution Operators (COs) and parallel computations with zero-gating on weights is used to compute in a 16-tiles per on-chip memory cycle. IFM is stationary for all the tiles which allows load-balancing with ease. This implementation with 14 nm accomplished a peak performance and energy efficiency of 256 × 10^9^ Operations/Second (OPS) and 15 × 10^12^ OPS/Watt per FC (VGG-16) layer respectively. Also, it improves energy efficiency to a maximum of 6.08 times and area efficiency to 7.6 times compared to the existing processors.

## Introduction

In recent years Deep Learning (DL) based Convolutional neural networks (CNN) have established significant advantages in computer vision domains, especially in classification, recognition, and segmentation. The CNN models such as AlexNet, VGGNets, GoogleNets, and ResNets have evolved for visual data processing applications in the field of agriculture, medicine, automotive, industrial, etc^1–3,33^. An appropriate CNN model with an adequate training dataset will lead to proper outcomes in a specific task, which is even better than manual inspection^2^.

**Fig. 1:**
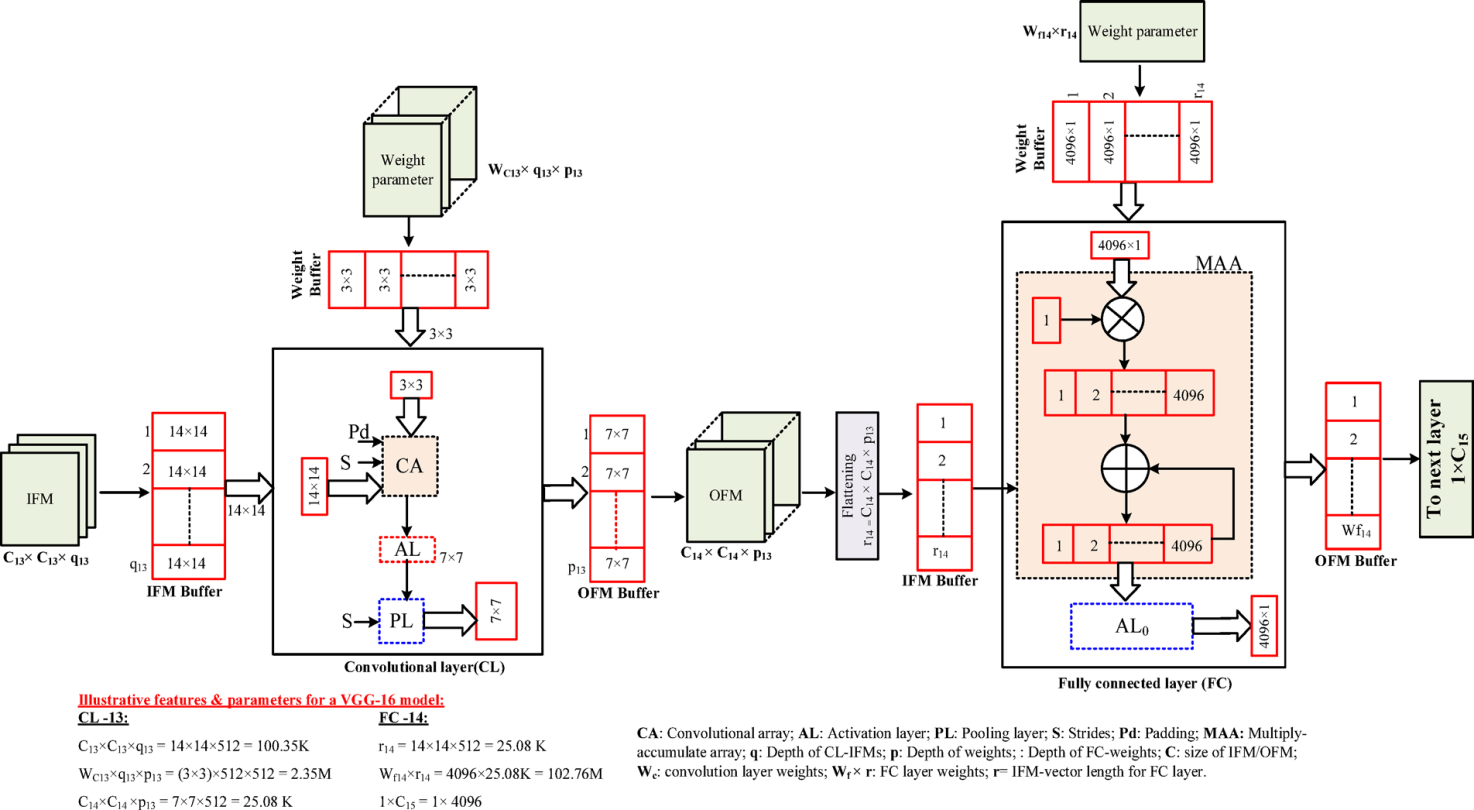
Data flow structure of 13th layer (CL −13) and 14th layer (FC – 14) of VGG − 16.

The general structure of the CNN model adopts a specific number of Convolutional Layers (CL) followed by an activation layer (AL), pooling layer (PL), and output classification at the Fully Connected layer (FC). Each CL and FC layer contains a stack of Convolution Operator (CO)/Multiply-Accumulate (MAC) computation. It extracts the feature map using its corresponding pre-trained filters/weights as shown in Fig. 1. In general, the layers of the deeper CNN models always hold millions of unique parameters (2D-weights) which provide better performance in output classification.

However, there are still huge challenges in the CNN model regarding the device’s processing speed and memory allocation due to its high computational and storage complexity^4,5^. For example, the most common automotive application domain of intelligent cars and unmanned aerial vehicles adopted the most popular deep learning models, which have a huge requirement for real-time systems. Meanwhile, the hardware constraints of edge-based devices demand improvements in the scalability and energy efficiency of deep learning models. Designing an optimized architecture for the deployment of CNN is therefore important in computer vision applications^6^.

The CNN inference can be implemented in the three most popular methodologies for embedded applications such as (1) Application Specific Integrated Circuits (ASIC), (2) Field Programmable Gate Arrays (FPGAs), and (3) Graphical Processing Units (GPUs). GPUs are extensively used in model training and inference. It provides high computing power and bandwidth. The high-power dissipation of GPU also restricts its application in embedded systems^6^. ASIC and FPGA are suitable for inference engine on embedded systems since it combines high efficiency, flexibility, and low power consumption^7,8^. However, the huge amount of computation and on-chip memory limit the performance of ASIC based CNN model^7^. Various deep learning (DL) based hardware acceleration papers have been published in the past decades, focusing on optimizing the computational complexity and memory requirements for the CNN models^9–14^.

The convolutional layer and fully connected layers exhibit different levels of complexity in terms of data flow, memory management, and computations. To explore these facts, a specific case of 13th layer (CL −13) and 14th layer (FC – 14) of VGG − 16 is illustrated in Fig. 1. Here, in the CL −13, a 14 × 14 IFM-frame from the IFM buffer and a 3 × 3 filter weights are supplied to the Convolutional Array (CA). The CA processes the IFM frame as 3 × 3 windowing with the stride (S) value given and necessary padding (Pd). After this activation and pooling layer produces 7 × 7 OFM-frames. These OFM frames are then flattened and stored in the IFM buffer of FC – 14. Each IFM and a corresponding set of weights (4096 × 1) are supplied to the multiply-accumulate array (MAA). Further, all IFMs are processed similarly and accumulated till they complete the IFM buffer. Finally, the set of OFMs (4096 × 1) is produced after the activation process.

From the above observation, the memory bandwidth for the FC layer is very huge compared to the CL layer. Also, there are more chances (around 80%) to have zero-valued IFMs, if the RELU activation function is used in the previous layers. In this case, the memory bandwidth for the FC layer can be drastically reduced if we use a suitable sparsity-based control strategy between the IFM buffer and MAA. Similarly, there have been considerable zero-valued IFMs are present in the CL layers. This has been addressed by many researchers^10–15^ and they proposed: (1) zero skipping^10,12^; (2) zero gating^13,14^; and (3) frame-wise compression^11,15,16^. A dedicated controller for detecting zero-valued IFMs and skipping it from the convolution operation was proposed in^32^, through this 2.45 times of energy efficiency was improved.

Therefore, different control strategies should be adopted for the fully connected layers and convolutional layers. Since only a few researchers^9,15^ explored strategies for the fully connected layers, this work concentrates on the fully connected layers’ operation and memory transactions for improving the energy efficiency of the CNN-based image classifier.

In response to the above understanding, this paper proposes a framework for accelerating and deploying ASIC-based accelerators for FC layers using compression techniques (zero skipping & gating) and memory optimization strategies. These techniques involved accelerators which have acceptable accuracy loss and high energy efficiency, making them suitable for running processors with low power consumption. The following are the contributions made in this work:

1. Sparsity is induced on pretrained FC layers weights ($\:{W}_{f}$), using threshold analysis on the standard pretrained CNN models for a specific sparsity level$\:\left(\eta\:\right)$. Through this process, sparsity is enhanced by making an additional > 20% of the pre-trained weights to be zero. The acceptable induced sparsity is validated based on the classification metrics: Accuracy (above 95%) and F1-score (above 0.96) of the resulting models compared to the baseline model.
2. The data (IFM and its associated weights) is arranged in the DRAM and SRAM in such a way that these can be accessed row-wise. The first column of each row has an IFM, followed by a corresponding set of weights. This helps to maximize the effectiveness of the whole architecture, in terms of simple indexing and load-balancing control.
3. A novel compression method, **C**ombined $\:\mathbf{I}\text{F}\text{M}$ & **W**eights - **Z**ero **V**alued **C**ompression (CIW-ZVC) is proposed for optimizing the data flow from off-chip DRAM to on-chip SRAM. CIW-ZVC identifies the zero-valued IFM using the MSB of the IFM, later only the Valid IFM (non-zero) and its associated weights are transferred to SRAM. This minimizes the data movement rate by 3.3 times and requires an SRAM size of 137 KBs.
4. The on-chip processor is designed with an array of 256-COs along with zero gating on zero-valued weights and involves tile-based processing. This accomplishes reduced power consumption by a factor of 1.18 to 2.94. The overall proposed accelerator achieves an energy efficiency of 1.62 times to 6.08 times per layer in the VGG-16 model. The proposed accelerator with CIW-ZVC is realized under 14 nm FinFET technology libraries and compared with the baseline of both structured/unstructured sparse accelerators^10–12,15,27^.

The paper is organized as follows: Notable research works on the sparsity-based accelerators and the compression methods used in each are presented in Section II. Error insertion approach-based classification metrics analysis and Induced Sparsity (IS) mechanism on the pretrained CNN models are introduced in Section III. The overall framework design flow, including the proposed CIW-ZVC unit and architecture of the proposed accelerator, is elaborated in Section IV. The storage policy and the acceleration method of the computing module are also emphasized in Section IV. The performance of the architecture and analysis are presented in Section V. The conclusions of this paper are presented in Section VI.

## Related works for sparsity-based CNN accelerators

An efficient hardware acceleration in CNN-based inference engines focuses primarily on the following aspects: the use of lower-precision data formats for weights & IFMs representation, introducing optimum dataflow scheduling, optimized on-chip memory, and implementation of effective pipeline/parallel architectures in processing elements (PEs). In the recent past, accelerators have exploited the sparsity in the input data, and different strategies to compress the data were presented in^9–16,34,35^. From these works it can be concluded that sparsity-based networks can provide benefits in terms of minimizing the computation & memory access and maximizing the effectiveness of physical characteristics in the overall hardware during inference.

From 2016 onwards, different methods of handling the zero-valued sparsity-based accelerator have been presented. KOP3^12^, Eyeriss^13^ accelerator utilizes the zero/clock gating approach based on the zero-flag in IFMs at the CL layer. KOP3 presented zero-gating specifically for the 3 × 3 convolution, whereas Eyeriss is designed for row-stationary MACs^12,13^. Moreover, it uses the 8b-FX precision and degrades the AlexNet and VGG-16 accuracy by 2% to 5% compared to the 16b-FX. The processor utilized the energy efficiency of 3.14 TOP/W, 65 nm technology in 1.3 V^12^. The Cambricon series in^14^ exploit the sparsity of weights for CL layer and FC layer computation where coarse-grained pruning is used to reduce the irregularity of sparse weights. But it affects the overall classification accuracy in the CNN model. EIE^9^ uses a compressed sparse column (CSC) approach in the FC layers. It adopts the relative row index and column pointer to locate the valid IFMs and weights for further computation in the on-chip processor. Also, the processor skips the invalid data from the computation in the FC layers of AlexNet. This approach is used for processing the samples at 1.88 × 104 frames/sec and it results in power dissipation of 600mW in the 45 nm implementation.

NullHop^10^ compresses the valid IFMs of each kernel in the convolution layer. It uses a sparsity map (SM) and a nonzero value list (NZVL) to decode and match the IFM and weights. It can support different kernel sizes which are required in VGG-16 & 19, Giga1Net, and RoshamboNet. This compression achieves an energy efficiency of 114.24% in the case of the VGG16 model for the ImageNet dataset of 1000 classes.

SNAP^11^ compresses both IFM and weights using an Associative Index Matching (AIM), it adopts the channel-based sparsity indexing approach. IFM data has been compressed based on the channel index of the corresponding valid data. Weight compression has based on the channel index, row index, and kernel index as well as position pointer. This approach maintains high hardware utilization and low writeback data traffic at the partial sums. It supports the CL layer, Point-Wise Convolution (PWC) layer, and FC layers. SNAP offers energy efficiency of 3.61 TOP/W for ResNet-50 in 16 nm.

Sparsity-aware hardware accelerator^15^ uses two symbol Huffman coding, similar to the zero-valued compression (ZVC) approach on the IFMs and weights. Huffman coding approach includes a flag bit, 1 denotes the valid data. This technique can support multi-function layers in CNN and RNN operations including CL, DWC, PWC, FC, and LSTM. This method achieves the performance of 102 GOP/s with a power consumption of 194mW for VGG-16 under 40 nm technology.

Video application-based accelerator^16^ adopts the channel run length (RL) and coordinate index to skip empty channels and zeros in intra-kernel. Before processing, the controller reads the Channel-RL and Word Numbering (WordNum) in the SRAM weights. Channel RL is used to determine the address space in the table mapping. WordNum signifies the number of nonzeros in the weight plane. It supports the CL, DWC, and PWC layers in the CNN models. Also, the architecture involves hybrid-based precision of 4 to 8 bits with an inter-frame-reuse approach which takes benefit of both low bit-width and high sparsity of differential frame data during the training. The processor provides energy efficiency of 0.129 TOP/W to 13.3 TOP/W for MobileNet under the 65 nm technology node.

Compression methods vary with respect to the deep-learning model, processing layer (CL, De-CL, DWC, PWC, FC, LSTM), memory available/required, and specific to the data (IFM/weight)^11,15^. Compression techniques have been adopted for the deep-learning model (CNN/RNN) based implementation which uses for both 2D (image) and 1D (speech) oriented data. Compression in the FC layer has more relevance than any other layers because of the chances of the majority number of zero-valued IFMs being produced by the convolutional layers before^9,11,15^. Section III elaborates on this and around 70% of IFMs could be zero-valued. The compression methods in^10,11,14,16^ are related to the memory overhead for indexing and load balancing. The complexity of these operations is based on the depth of the layers and sparsity on IFM^10,11^/weights^11,14,16^.

**Fig. 2:**
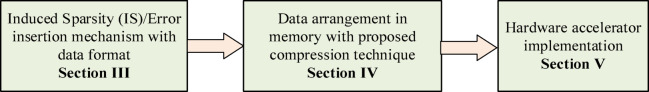
An overview of chapters.

Considering the above-related works, this paper attempts to implement a customized compression technique specifically for the FC layer of the CNN-based IEs. The proposed hardware accelerator controls the data flow from off-chip DRAM to on-chip SRAM. After checking the validity of the IFM, only non-zero IFMs and their associated weights are pushed in the SRAM. This will reduce the read and write cycles in both memories. Also, the size of the on-chip SRAM is reduced considerably. This is well suited for the unstructured zero sparse models. Further, a software-based induced sparsity has been introduced on the pretrained FC layer weights to reduce the computation complexity in the accelerator without affecting the classification metrics. Particularly, the proposed hardware accelerator concentrates more on the data arrangement and optimization of the data flow to the processor array. Fig. 2 illustrates the Section connections overview.

## Software-based induced sparsity (IS) mechanism in CNN pretrained models

### Performance analysis of induced sparsity (IS) in pretrained weights $\:{(\varvec{W}}_{\varvec{f}})$ for CNN models

The performance, classification accuracy, and cost are the prime factors in CNN-based pre-trained inference engines. These factors depend on the number of Convolution Operators (CO)/Multiply-Accumulate (MAC) unit computations required per model and the optimal data format used in it. Each CO unit accepts two inputs, an input feature map, and a pretrained weight. Typically, a considerable number of IFMs are turned to zero due to ReLU, (If $\:IFM\le\:0$, then $\:OFM=0$ else *O*$\:FM=IFM$) in each activation layer (AL) after the Convolutional Layer (CL). As there are multiple convolutional layers before the data reaches fully connected layers, the majority of the IFMs are turned to zero. To study this further, fully connected layers of three standard CNN models as given in Table 1 are considered. The standard pretrained CNN models (such as AlexNet, VGG-16, & VGG-19) have been taken for analysis using the ImageNet dataset for training the model^19^. For worst-case analysis, each model adopted the classes of 1000 at the output classification. The IFM sparsity ratio ($\:{S}_{IFM}$) has been calculated for each FC layer in all the CNN models with random test images using a MATLAB-based environment. It is clear from Table 1 that, on an average 77% to 80% of IFMs in the FC layers are zero-valued.

**Table 1:** IFM sparsity (S_IFM_) IN FC layers of the standard cnn models

Layer	$\:{\mathbf{S}}_{\mathbf{I}\mathbf{F}\mathbf{M}}=\frac{\mathbf{N}\mathbf{u}\mathbf{m}\mathbf{b}\mathbf{e}\mathbf{r}\:\mathbf{o}\mathbf{f}\:\mathbf{z}\mathbf{e}\mathbf{r}\mathbf{o}\:\mathbf{v}\mathbf{a}\mathbf{l}\mathbf{u}\mathbf{e}\mathbf{d}\:\mathbf{I}\mathbf{F}\mathbf{M}\mathbf{s}}{\mathbf{T}\mathbf{o}\mathbf{t}\mathbf{a}\mathbf{l}\:\mathbf{n}\mathbf{u}\mathbf{m}\mathbf{b}\mathbf{e}\mathbf{r}\:\mathbf{o}\mathbf{f}\:\mathbf{I}\mathbf{F}\mathbf{M}\mathbf{s}\:\mathbf{p}\mathbf{e}\mathbf{r}\:\mathbf{F}\mathbf{C}\:\mathbf{l}\mathbf{a}\mathbf{y}\mathbf{e}\mathbf{r}}\times\:100$		
	AlexNet	VGG-16	VGG-19
FC-1	69.30	87.23	86.42
FC-2	85.40	82.11	80.13
FC-3	78.91	70.54	74.61
**Average**	**77.87**	**79.96**	**80.38**

Along with these zero-valued IFMs, a considerable set of weights can be eliminated from the computation. But, the complexity of FC layers is still not reduced due to the large volume of weights being used in the MAC operations. Therefore, the insignificant pre-trained weights of the fully connected layers ($\:{W}_{f}$) have to be identified and converted to zero-valued weights without affecting the required classification metrics such as accuracy and F1-Score. This process is called induced sparsity on $\:{W}_{f}$.

There have been many previous works which focused on the different methods to for inducing sparsity in training^20^ and testing/inferencing^21^. Here in this work, the threshold based induced sparsity on inferencing is considered. Selection of threshold value among $\:{W}_{f}$ have directly impacted the classification metrics drastically. In most of the standard threshold-based works, a $\:\pm\:\:threshold\:value\left(\varDelta\:\right)$ chosen, and the $\:{W}_{f}$ values that fall in this range are converted into zero-valued weights $\:{W}_{f0}$. Therefore, the set of pretrained fully connected weights ($\:{W}_{f}$) are now converted into a new set $\:\left(Wg\right)$ which has both zero-valued weights $\:{W}_{f0}$ and non-zero-valued weights $\:{W}_{fN}$.

**Fig. 3:**
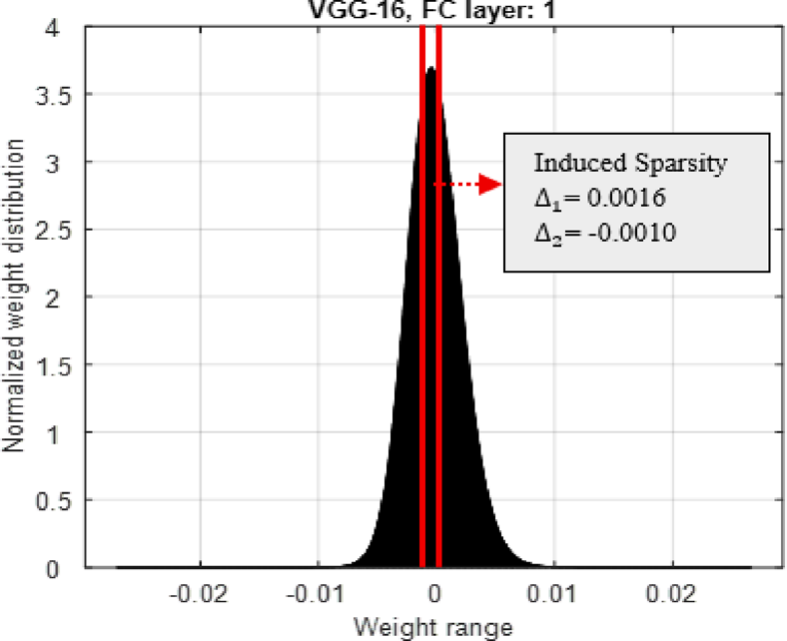
Distribution of pretrained weights for 1000 classes- a layer of FC for the VGG-16 model. (Induced the zero sparsity between the lines).

**Fig. 4:**
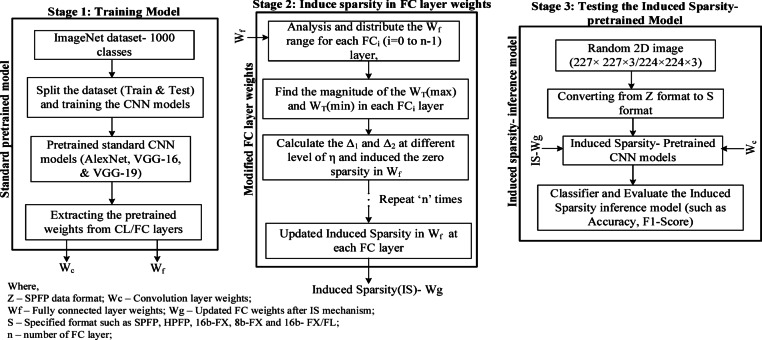
Mechanism of Induced Sparsity (IS) in pretrained FC layer weights $\:{(W}_{f})$.

The histogram of the $\:{W}_{f}$ -values in the fully-connected 1 st layers of the standard CNN models (VGG-16) are recorded as shown in Fig. 3. The distribution of $\:{W}_{f}$ in the ranges ($\:{W}_{fmin}\:to\:{W}_{fmax})$ and is different in each layer and model. The three-stage process flow is proposed here to fix the maximum threshold value (Δ_1_) and minimum threshold value (Δ_2_) as illustrated in Fig. 4.

**Table 2:** Percentage of zeros induced, S_wg (%) for different sparsity levelS η

FC layers	VGG-16				
$\:\varvec{\eta\:}$	**5**	**10**	**15**	**20**	**25**
$\:{S}_{wg}\left(\%\right)=\frac{\text{N}\text{u}\text{m}\text{b}\text{e}\text{r}\:\text{o}\text{f}\:\text{z}\text{e}\text{r}\text{o}\:\text{v}\text{a}\text{l}\text{u}\text{e}\text{d}\:\text{w}\text{e}\text{i}\text{g}\text{h}\text{t}\text{s}}{\text{T}\text{o}\text{t}\text{a}\text{l}\:\text{n}\text{u}\text{m}\text{b}\text{e}\text{r}\:\text{o}\text{f}\:\text{w}\text{e}\text{i}\text{g}\text{h}\text{t}\text{s}\:\text{p}\text{e}\text{r}\:\text{F}\text{C}\:\text{l}\text{a}\text{y}\text{e}\text{r}}\times\:100$	13	23.9	31.2	42.1	54.2

Stage 1 is to get the baseline weights ($\:{W}_{c}$, $\:{W}_{f})$. Stage 2 is the process to induce sparsity in the $\:{W}_{f}$ based on sparsity level, $\:\eta\:$. Considering an induced sparsity level, random values of the maximum threshold value (Δ_1_) and minimum threshold value (Δ_2_) are fixed as given below:.

1 $$\:{\varDelta\:}_{1}=\frac{{W}_{fmax}\times\:{\upeta\:}}{100},\:\:{\varDelta\:}_{2}=\frac{{W}_{fmin}\times\:{\upeta\:}}{100}$$

The same will be repeated 3 times as there are 3 fully connected layers in the considered standard CNN models. For each sparsity level $\:\eta\:$, there is a certain percentage of zeros induced in the $\:{W}_{f}$, $\:{S}_{wg}\left(\%\right)$ in all 3 fully connected layers of the CNN models. $\:{S}_{wg}$ refers to the percentage of zeros induced in weights. At the end of this process a new set of weights for the FC layers $\:Wg$ is found. For example, at $\:\eta\:=5$, $\:{\varDelta\:}_{1}=\:0.0016$ and $\:{\varDelta\:}_{2}=\:-\:0.0010$ are estimated from $\:{W}_{fmin}=-0.0217\:\:to\:{W}_{fmax}=0.0326$ for the FC layer 2 of the VGG-16 and $\:{S}_{wg}\left(\%\right)=13$. Similarly, the percentage of zeros induced $\:{S}_{wg}\left(\%\right)$ for different sparsity levels, $\:\eta\:$ in each of the FC layers in VGG-16 models, and it is recorded in Table 2.

### Data format selection for processing the FC layer

**Fig. 5:**
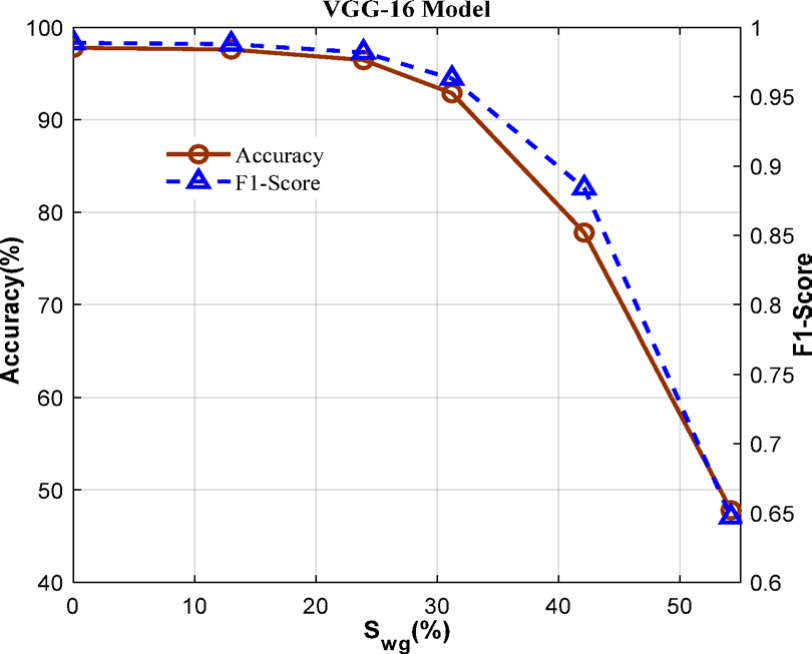
$\:{S}_{wg}\left(\%\right)$ vs. accuracy and F1 score for VGG-16.

**Fig. 6:**
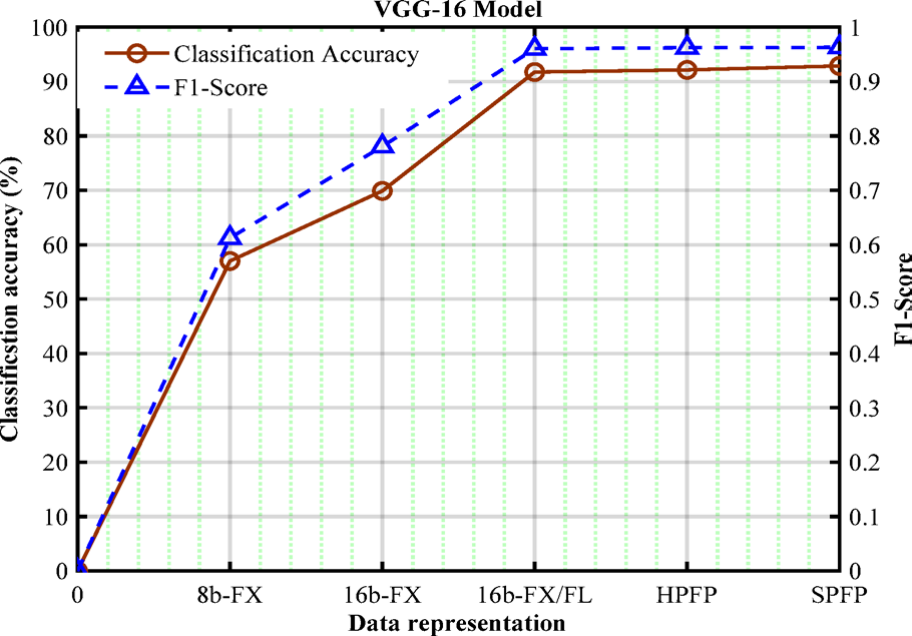
Accuracy and F1-Score vs. different data formats for VGG-16 model.

As per^22^, the minimum acceptable levels of accuracy and F1-score are 95% and 0.95, respectively. Therefore, from Fig. 5, the VGG-16 can have induced sparsity weights- $\:{S}_{wg}\left(\%\right)$ in the range of 22.8% to 27.7% while still achieving the required accuracy of 95% and F1-score of 0.95. The classification metrics also vary with respect to the data format adopted in the computation. With the above fixed induced sparsity levels, the model was analyzed for the best-suited data format among 8-bit Fixed point (8b-FX), 16-bit Fixed point (16b-FX), Half- precision floating point (HPFP), Single-precision floating point, and 16-bit mixed precision format (IFM: 16b-FX and Wg: HPFP) (16b-FX/FL)^22^ as shown in Fig. 6. Here, the baseline data format is considered as single precision floating point (SPFP). The analysis reveals that 16b-FX/FL can be chosen considering the accuracy and F1-Score for the standard CNN models. Also, 16b-FX/FL is marginally lesser than SPFP and nearly equal to the HPFP in terms of the above-mentioned metrics. Also, the 16b-FX/FL data format could be more beneficial in terms of hardware implementation compared to HPFP.

The optimum data format representation for IFM and $\:Wg$ is significant in optimizing the hardware implementation. IFM-based $\:Wg$ movement from off-chip to on-chip memory reduces the CO computation, on-chip storage requirements, and thereby hardware cost. Furthermore, with an induced sparsity mechanism on $\:{W}_{f}$ and in turn $\:Wg\:$improves hardware efficiency and performance, without affecting the classification accuracy. However, handling the $\:{S}_{IFM}$ and $\:{S}_{wg}$ requires extra control modules for load balancing and indexing to process the skipping, compression, and gating.

**Fig. 7:**
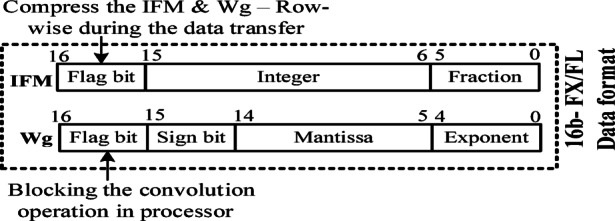
Data format representation for $\:\varvec{I}\varvec{F}\varvec{M}$ and $\:\varvec{W}\varvec{g}$.

The FC layers in the CNN model have to process unstructured sparse zero-valued IFMs and weights$\:\:\left(Wg\right)$. A specific set of $\:Wg$ is converted into a zero-valued by the Induced-Sparsity mechanism. Also, the suitable data format to represent IFMs and corresponding weights $\:\left(Wg\right)$ is selected as explained above. The adopted data format with a Flag bit as MSB to compress the IFM and Wg are shown in Fig. 7. Accordingly, the zeros in the unstructured sparse FC layer’s IFM (around 77% to 80%) and weight, $\:Wg$ (10–30) % can be compressed effectively.

Considering these factors, the hardware accelerator has been proposed to optimize the complex modules for the unstructured sparse model as discussed in Section IV.

## Hardware accelerator for sparsity-based CNN model

### Data arrangement and analysis of memory requirements

**Fig. 8:**
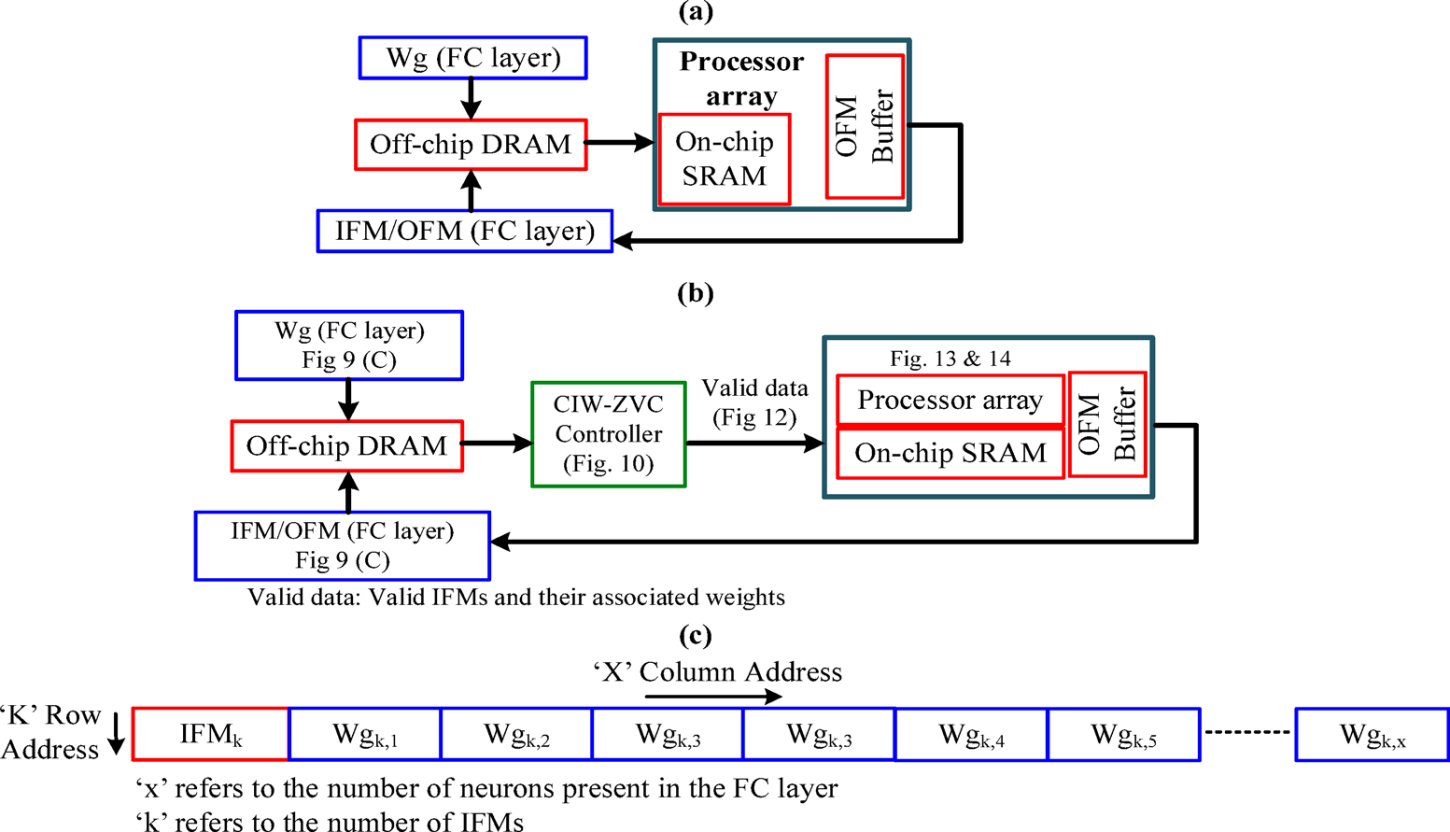
**(a)** A general structure, **(b)** Controller based on the FC Layer processing scheme, **(c)** Row-wise data arrangement in RAM.

There have been many techniques and strategies were reported to effectively manage this zero sparsity^23^. Most of the previous works adopt (1) Zero skipping; (2) Zero gating and (3) combined skip-gate at either only on IFMs or weights or both. Also, all these strategies are implemented along with the processor array on an IC package. The required IFMs and corresponding $\:Wg\:$are supplied from an external DRAM to the on-chip SRAM as shown in Fig. 8(a). If an IFM is zero-valued, its corresponding weights (considerably large volume) need not move to on-chip SRAM.

Therefore, a controller checks whether the IFMs either zero or not and then forwards the corresponding set of weights accordingly as shown in Fig. 8(b). Also, the data is proposed to be arranged specially in DRAM and SRAM. All IFMs have been kept in the first column of each row and respective weights are in the other columns of the same row. The data arrangement is shown in Fig. 8(c). This strategy surely will reduce the data movement rate between DRAM to SRAM. Also, it reduces the size of SRAM and maximizes the utilization of the MAC operators in the processor array.

**Fig. 9:**
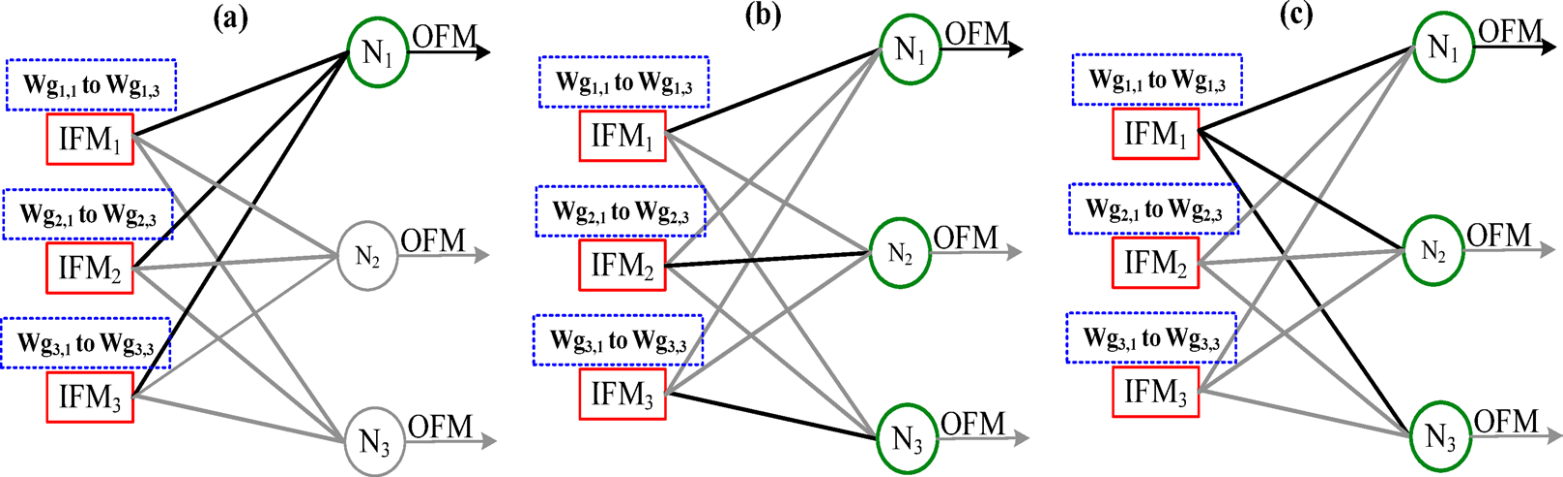
Input data processing in the FC layer. **(a)** IFM-stationary with neuron-wise computation, **(b)** Network adopted for $\:IFM$ and $\:Wg$ are non stationary structures, **(c)** $\:IFM$-stationary and $\:Wg$ are non-stationary structures.

There are different strategies for processing the input in the FC layer as shown in Fig. 9. These strategies focus on optimizing the memory transactions. IFM non-stationary and neuron-wise computation, as shown in Fig. 9(a) could be more suitable for word-serial architecture^5^. The strategy illustrated in Fig. 9(b) is for parallel architecture^9^. These structures need an additional controller for load balancing (specific IFM goes with corresponding $\:Wg$) in an unstructured sparse FC layer. A stationary IFM and parallel data flow, as in Fig. 9(c) is proposed to optimize the on-chip memory requirement. The sparsity in the IFMs is taken care of in the data path between DRAM to SRAM and reduces considerable on-chip memory. Zero-gating is applied with respect to zero-valued $\:Wg$. Therefore, a simple controller is sufficient, and it leads to low power consumption.

### Combined IFM and weights - zero value compression (CIW-ZVC)

**Fig. 10:**
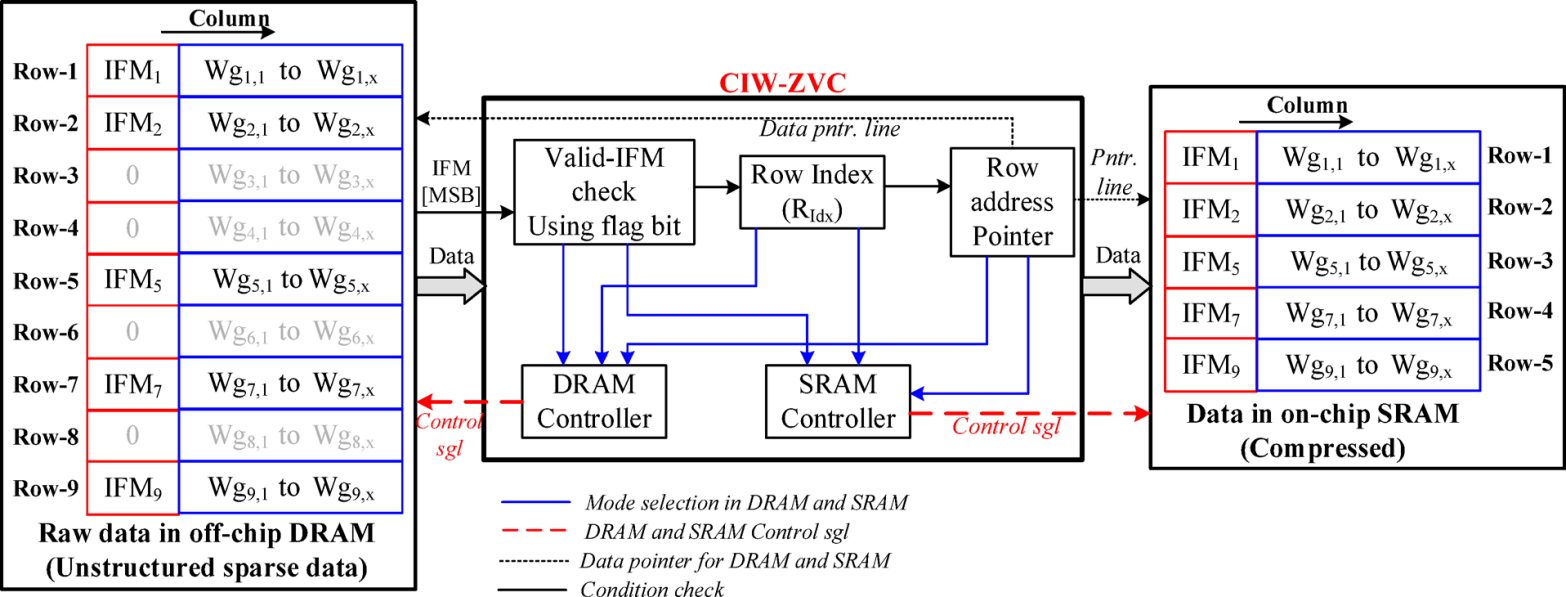
Raw data compression using CIW-ZVC controller between DRAM and SRAM.

Considering the strategy presented in Fig. 9(c) and the transfer of only valid (non-zero valued) IFM and its corresponding weights to the processor array using proposed CIW-ZVC as shown in Fig. 10, the off-chip DRAM is to store the raw IFMs and pretrained weights (both$\:\:IFM$ and $\:Wg$ are an unstructured sparse manner) of the CNN model. The on-chip SRAM is to have compressed IFMs and their associated array of weights ($\:Wg$- unstructured sparse).

There are three standard compression methods were proposed in the earlier works^23,24^, such as (1) Zero value compression (ZVC), (2) Run-length encoding (RLE), and (3) Compressed sparse row (CSR). These compression techniques require additional storage for indexing and load balancing with respect to IFM and Wg. Moreover, these methods require additional hardware overhead due to complex control strategies implemented in the processor array. From these compression methods, ZVC has adopted an additional bit (1-for Zero valued) as MSB along with the absolute value of IFM/Wg. This additional bit is used for easy indexing of all non-zero valued IFM and Wg, but it makes load balancing (among IFM and corresponding Wg) more complicated in the FC layer processing. The RLE maintains the number of zeros present between two consecutive non-zero values to make the load-balancing strategy simple. But this increases memory requirement and processing delay. The CSR is similar to RLE but it is for 2-dimensional memory structure (row and column-wise data). Considering these factors, a modified and efficient strategy, Combined IFM & Wg- Zero Valued Compression (CIW-ZVC) is proposed for processing FC layers in this work as shown in Fig. 10.

Similar to ZVC, the CIW-ZVC method uses an 8-bit row index (R_idx_) and an additional flag (MSB) bit in IFMs. This MSB bit is useful in identifying and skipping the zero-valued IFM and weights. If a zero-valued IFM is found, then all its associated Wg will be skipped in moving and storing the compressed data to the on-chip SRAM. The row address pointer helps to point the position of the data for reading and writing from DRAM to SRAM write using the mode selection. The model selection of the DRAM and SRAM controller has been discussed in Section IV. C. For example, If the 17th bit of IFM_3_ is 1, the IFM_3_ value and associate WG_3_= {Wg_3,1_, Wg_3,2_,., Wg_3,x_} has to be skipped from the row buffer. Otherwise, the non-zero valued IFM and associated weights Wg are to be moved to SRAM for further processing.

Table 3 illustrates the expression to calculate the memory requirement for the standard compression scheme and proposed CIW-ZVC. Where $\:{\mathbf{N}\mathbf{Z}}_{\mathbf{v}}$ – Number of non-zero values; $\:{\mathbf{Z}}_{\mathbf{v}}\:$– Number of zero values; **L** – One-bit binary index/flag (‘1 – Zero valued or 0 – Non-zero-valued data); **K** – Number of bits used to represent the IFMs & weights; **M** – Maximum number of bits required to address the $\:{\text{N}\text{Z}}_{\text{v}}$ of previous rows in each row (row pointer); **N** – Maximum number of bits required to count $\:{\text{Z}}_{\text{v}}\:$before the $\:{\text{N}\text{Z}}_{\text{v}}$ in each row (column index); **P** – Maximum number of bits required to count $\:{\text{Z}}_{\text{v}}$ between the $\:{\text{N}\text{Z}}_{\text{v}}$ (run length index); **R**_**idx**_ – Maximum number of bits required to address the rows (row index). The memory requirement on the compression schemes depends on the non-zero values ($\:{NZ}_{v})$ with their data representation and capacity of the indexing.

**Fig. 11:**
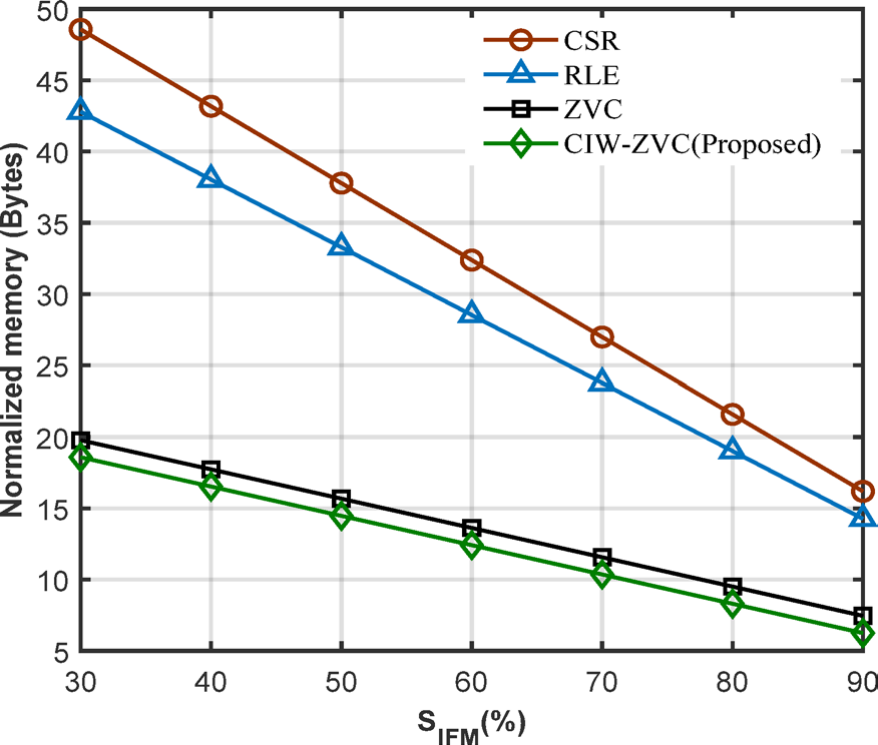
Memory requirement for standard compression schemes at different levels of S_IFM_.

Here, assume that the size of the FC layer is I = 256 (H) × 4097 (W) (1st column: IFM & 2nd to 4097th column: Wg) for the analysis of different compression schemes. The memory requirement vs. different levels of sparsity for compression methods are plotted in Fig. 11. From the plot, the proposed CIW-ZVC reduces the memory capacity by 1.08 times, 2.2 times, and 2.6 times compared with ZVC, RLE, and CSR respectively. CIW-ZVC gives flexibility during the data movement and reduces the complexity of indexing techniques, especially for FC layers.

**Table 3:** Expressions to calculate memory requirement for different compression schemes add columns.

Compression	Memory required (in bits)	Indexing allocation
ZVC	$\:{(NZ}_{v}\times\:K\:bit)+\left(I\times\:L\:bit\right)$	$\:{NZ}_{v}$+ binary index value
CSR	$\:{(NZ}_{v}\times\:K\:bit)+{(NZ}_{v}\times\:N\:bit)+(H\times\:M\:bit)$	$\:{NZ}_{v}$+ column index + row pointer
RLE	$\:{(NZ}_{v}\times\:K\:bit)+({NZ}_{v}\times\:P\:bit)$	$\:{NZ}_{v}$+ run length index
**Proposed CIW-ZVC**	$\:({NZ}_{v}\times\:K\:bit)+H(L\:bit+{R}_{idx}\:bit)$	$\:{NZ}_{v}$+ binary index + row index

Note: L = 1b; K = 16b; N & M = 13b, *P* = 21b; R_idx_=8b for the assumed data size of I = 256 × 4097 from the part of an FC layer in VGG-16.

### Compressed dataflow between the DRAM, SRAM, and processor array

**Fig. 12:**
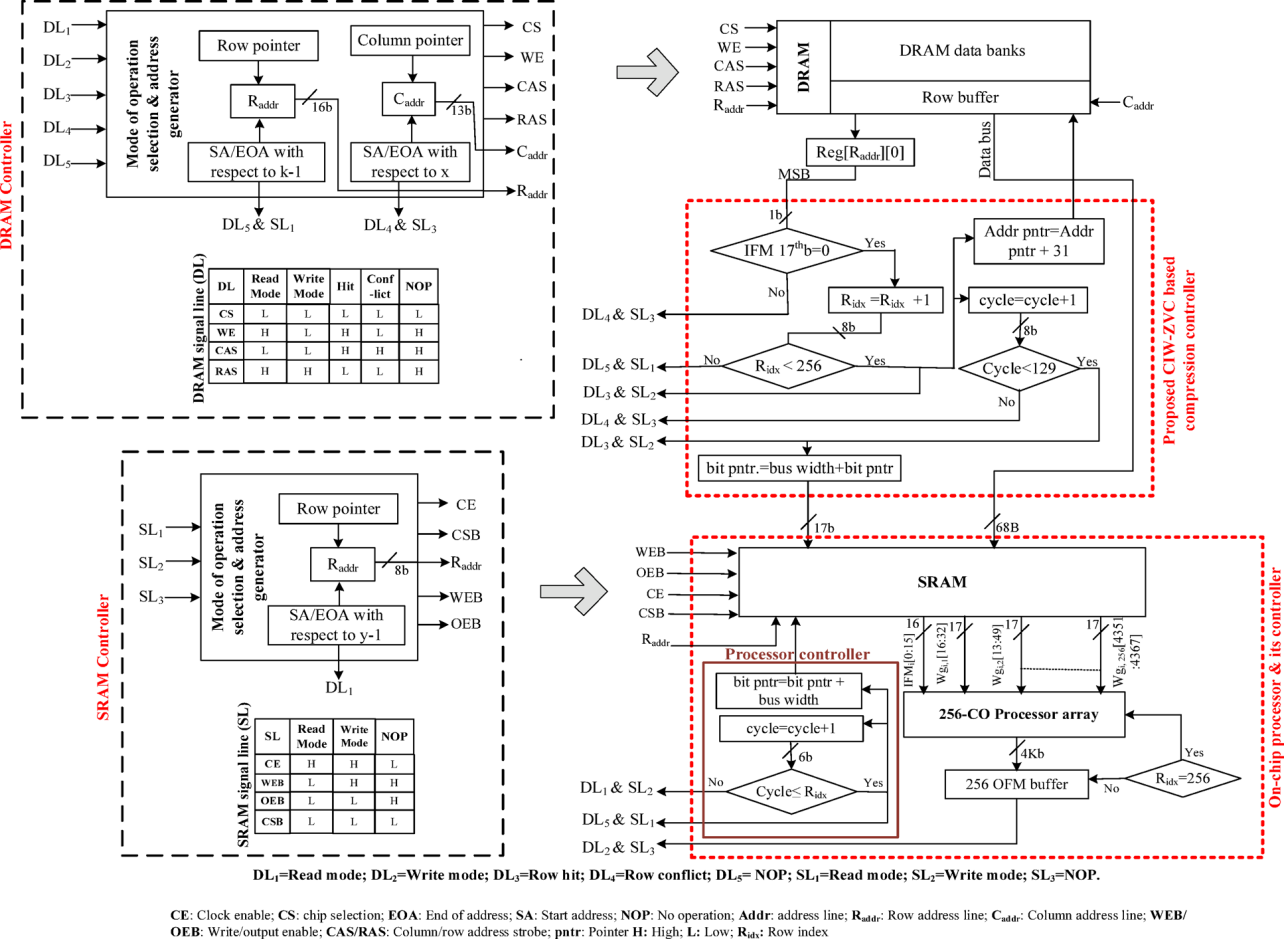
Detailed structure of off-chip to on-chip data movement using proposed CIW-ZVC.

An efficient memory controller is proposed, to compress the IFMs and their associated weights as shown in Fig. 10. This controller uses the proposed CIW-ZVC for compression and later it transfers only the valid data from DRAM (off-chip) to SRAM (on-chip), and also skips the invalid data. The detailed architecture of the proposed CIW-ZVC based memory controller is illustrated in Fig. 12. In this proposed design, three modules are involved for the data movement and data compression between the memories. CIW-ZVC based compression controller, read controller for DRAM and read/write for SRAM.

To have specifications for further design of the proposed controller, here the first fully connected layer of VGG − 16 has been considered for implementation. Therefore, in off-chip DRAM, the complete set of IFMs and associated Wg required for the processing of this layer is stored. Also, considering the proposed data formats (Section III.B) and the data arrangement (Fig. 8(c)), the capacity of DRAM has been finalized as 34.5 MB. Here, the processor array considered can process 256 IFMS and 256 weights concurrently. Therefore, the optimum SRAM size needs to be 140KB. The valid data transfer has been planned as per the capacities of both DRAM and SRAM. The valid data set in each row of the DRAM has been transferred to the row of SRAM. Similarly, 256 valid rows reach the SRAM in a particular number of DRAM-read and SRAM-write cycles.

The proposed memory controller starts the process by making the control line DL_1_ high, which sets the *read mode*. The *read mode* generates the row address based on the Starting Address (*SA)* and the row pointer. The generated row address enables the reading of the first row (IFM and corresponding Wg) from the data bank and pushes it to the row buffer. This transfer will happen in the *row conflict mode*, i.e., DL_4_ and SL_3_ are set to be high. Immediately after this, the column address has been initiated using *SA* and column pointer, to read each column of the row buffer. This data reading happens in the *row hit mode*, i.e., DL_3_ and SL_2_ are set to be high. Later, the data from the row buffer is transferred to the on-chip memory through the CIW-ZVC unit.

The CIW-ZVC unit analyses the data from the row buffer for the skipping and compression process. Further, it transfers the valid data to the on-chip SRAM. The CIW-ZVC unit receives the first word (considered to be 17-bit IFM) from the row buffer, and the MSB (17th) bit is taken to check either zero-valued IFM (1) or non-zero valued (0). If the MSB is 1, then entire row is skipped and the process proceeds with the reading of the next row from DRAM by activating the DL_4_ & SL_3_ and entering into the *row conflict mode*. Similarly, for valid IFM, i.e., MSB is 0, the entire data from the row buffer has to be pushed to a specific SRAM address. To check the space availability in SRAM, the condition *R*_*idx*_ *< 256* is examined.

If this is true, then DL_3_ & SL_2_ the *row hit mode is* activated, and the valid row is started moving from the row buffer to SRAM in batches. A row address pointer confirms the row in which this data is to be stored. A bus with 68 bytes capacity is used to do data transfer. The start and end bit address in that row is managed by the bit pointer of the SRAM. Each valid row of data in the row buffer requires a total of 129 bus cycles. After this, the memory controller reads the next row in the DRAM by activating DL_4_ & SL_3_ and therefore enters into *row conflict mode* and enables *NOP* in SRAM. Similarly, 256 rows of data are transferred from DRAM to the SRAM. After this, the SRAM has valid rows of 256 and 4096 weights in each row. Also, SRAM shifts from write mode to read mode by activating DL_5_ & SL_1_, and at the same time DRAM into *NOP mode.*

**Fig. 13:**
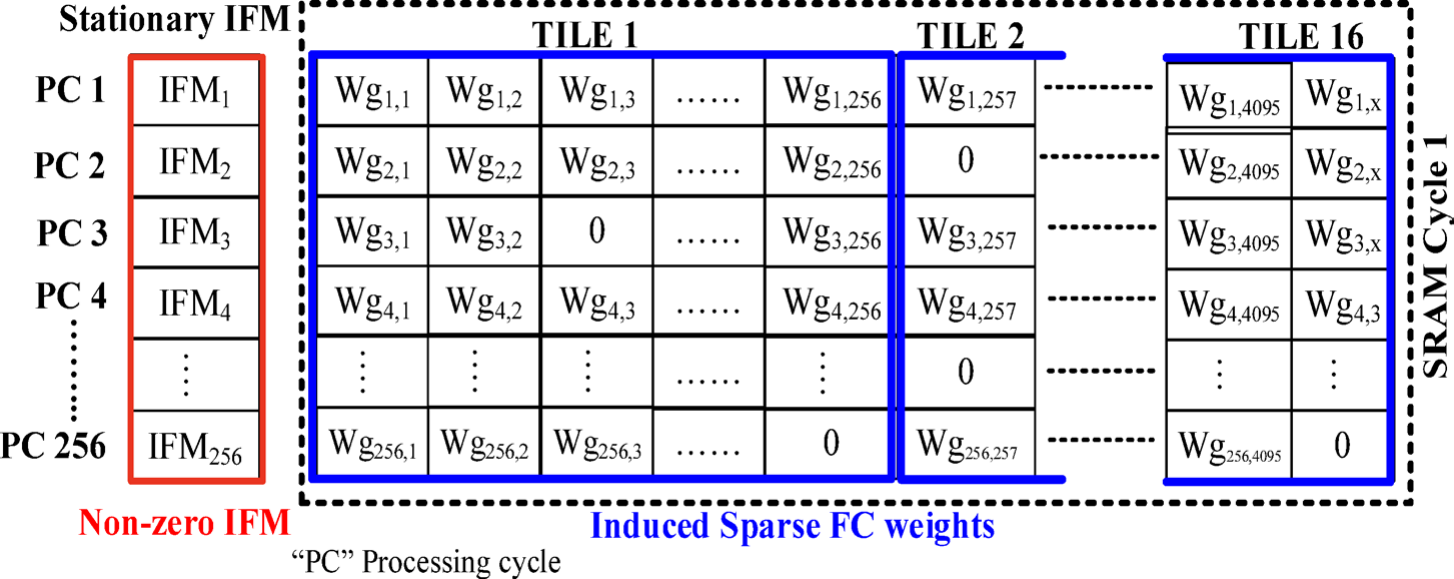
Tile-based arrangement of compressed IFM and Wg for an SRAM cycle. (Each tile: 256 IFM vector & 256 × 256 Wg matrix).

Now, the SRAM pushes the data in parts (a column vector of 256 IFMs and a tile of weights 256 × 256) to the processor array whenever the cycle condition is $\:cycle={R}_{idx}$. Therefore, 16 tiles are required to process the whole data in each SRAM cycle. The valid IFMs are stationary for all the tiles as shown in Fig. 13. Each tile has to be processed by a processor array, and it contains an array of concurrent 256 CO/MAC units.

**Fig. 14:**
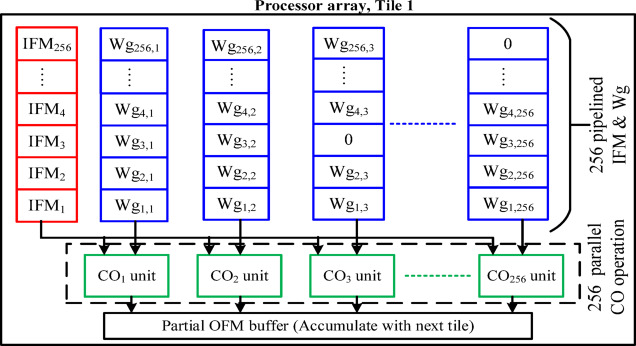
Data flow in an array of parallel 256 - CO/MAC units − 256 processor cycles per tile.

The CO/MAC unit uses the 16-bit FX/FL data format as given in Fig. 7 excluding the Flag bits (MSB). The array of CO units accepts one IFM and 256 Wg row-wise from the tile. The next IFM and set of weights (next row of 256 Wg) are pushed for processing based on the bit pointer in the SRAM controller. Therefore, each tile requires 256 processor cycles to complete the computation and store it in a set of partial OFM buffers (256 registers). This process is illustrated in Fig. 14.

**Fig. 15:**
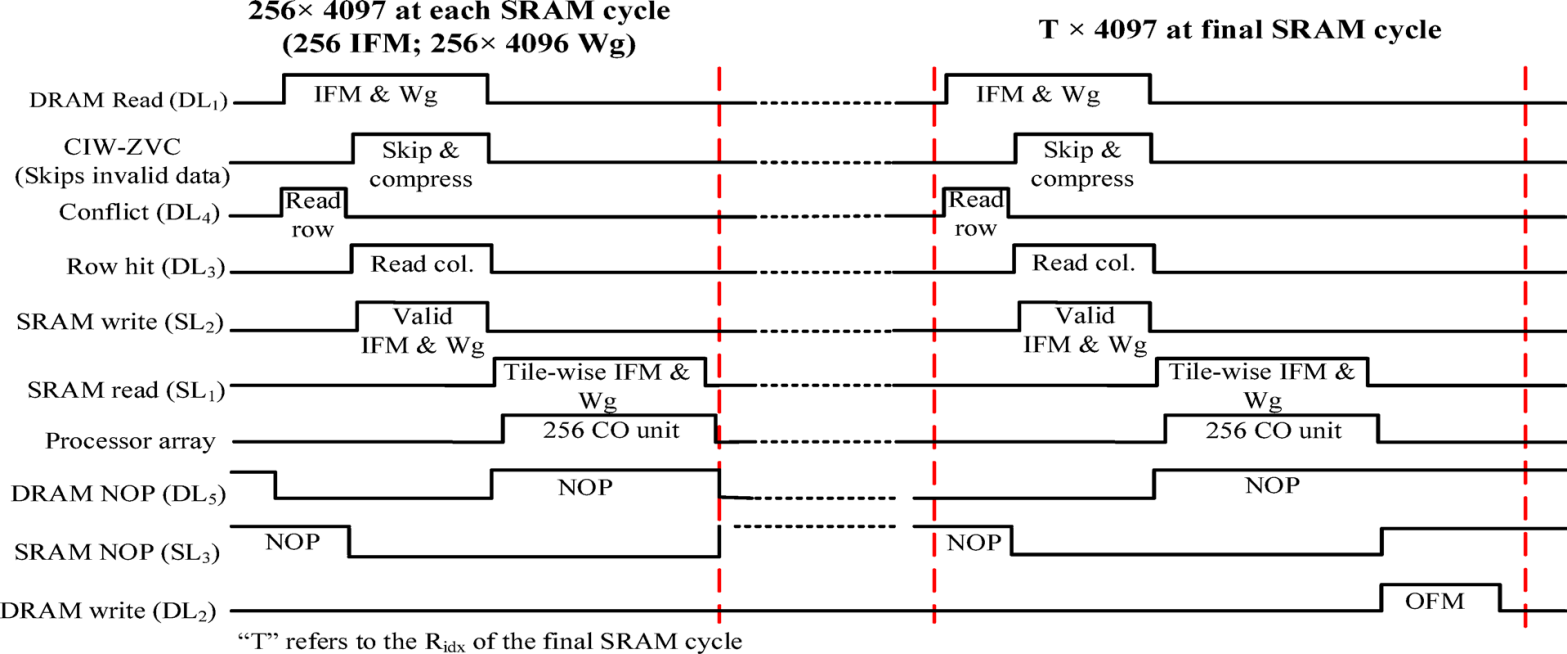
Synchronised read & write management between DRAM & SRAM.

Likewise, with the same 256 IFMs, 16 different tiles of weights are processed in the SRAM cycle and store the partially accumulated output in 16 different OFM buffers. Therefore, the partial 4096 OFMs will be available for tile-based accumulation in the subsequent SRAM cycles. Similarly, all the valid-IFMs are processed batch-wise as mentioned above. After every batch of the IFM process, DL_1_ activates the *read mode* in DRAM to read the data from DRAM and SL2 activates the SRAM to accept the data for the subsequent SRAM cycles. In the final IFM batch, there is a possibility of different data sizes (mostly lesser than or equal to 256 rows) due to the compression mechanism adopted in the CIW-ZVC controller. Therefore, zeros are padded to make the IFM batch 256, which will be processed in the final SRAM cycle. Consequently, the respective 4096 weights for each zero-valued IFM are also forced to be zero-valued. These zero-valued weights will be gated to skip the MAC operations. During this period, The CO units are in an idle computation state. Finally, the 4096 OFMs of this FC layer are written back to the DRAM data banks for further processing by activating the DL_1_ and SL_3_ as shown in Fig. 12. The synchronized data movement between DRAM and SRAM through the CIW-ZVC unit with necessary control signals is illustrated in Fig. 15.

Considering the VGG-16; FC-2 layer’s data movement requirement, the DRAM, SRAM, and CIW-ZVC controller are designed and synthesized using 14 nm technology libraries in the typical effort. The synthesis results of the CIW-ZVC unit are recorded in Table 4. This unit in the memory controller requires minimal hardware overhead. Despite this overhead in area and power, there is an appreciable improvement in data movement rate and latency in the processor array. The total latency and data movement rate is calculated for the DRAM, SRAM, and CIW-ZVC controller combination which processes the above said FC layer. Each row of data (one IFM and 4096-Wg) requires a total of 51µs to transfer between the memories. This data transfer happens through a 68 Byte data bus, and it requires 400ns for the transfer. Correspondingly, the SRAM cycle (transfer of 256 IFM and 256 × 4096 weights) needs 13ms to transfer the data from DRAM to SRAM. Compared with standard data movement (without CIW-ZVC), the proposed controller increases data movement rate 3.3 times and latency for 70% of S_IFM_ and 20% of S_Wg_, as given in Table 5. This range of sparsity is adopted from the software analysis discussed in Section III.

**Table 4:** Synthesis results for the CIW-ZVC implementation in hardware

CIW-ZVC unit	Area (µm^2^)	Power (µW)	Delay (ns)
	267.5	62.9953	1

**Table 5:** Data movement rate for the FC layer-2 in VGG-16 using CIW-ZVC

**VGG-16:** **FC layer-2 @** S_IFM_= 70% S_Wg_= 20%	**Total latency (ms)**		**Data movement rate**		**DRAM access** **(MB)**
	**Without** **CIW-ZVC**	**With CIW-ZVC**	**Without CIW-ZVC**	**With ** **CIW-ZVC**	
	209.7	62.4	164.2×10^6^	553×10^6^	34.5

Due to this process, the effectiveness of the SRAM and on-chip processor has been enhanced in the proposed accelerator by keeping only the valid IFMs and their accompanying Wg. For the assumed example, the standard design needs 256 SRAM cycles to process the complete data from the DRAM. Due to the proposed CIW-ZVC, only the valid data from DRAM are moved, and only 48 SRAM cycles are required to process. So, the storage of the SRAM has been reduced by 3.2 times. The proposed on-chip processor does not require an extra control unit for load balancing and sparsity indexing. The on-chip processor array accesses directly the data from the SRAM and processes it.

Furthermore, the power requirement for the proposed data access (from DRAM to SRAM through CIW-ZVC controller) has been estimated considering the following activities: (1) DRAM read; (2) SRAM write/read for the unstructured sparse model. The power requirement for these activities is estimated with specified sparsity levels (S_IFM_=70%, S_Wg_=20%) and using 14 nm libraries in a typical effort. Later it is compared with the standard data access model (absence of zero-valued skipping and gating) as listed in Table 6. The dynamic power in DRAM read activity is improved by 80.5% for 70% unstructured sparsity in IFM and its corresponding Wg. This is because these data are not pushed into the SRAM for further processing. In SRAM, the dynamic power has improved by 15.7%. This is due to the sparsity consideration of Wg being around 20%, (for maintaining the classification accuracy). Also, most of the invalid data are skipped in the CIW-ZVC controller during the data transaction. The static power in both activities is nearly similar in the standard model (without zero skipping and gating) and the proposed model. Considering the total power, the proposed accelerator achieves 42% less compared to the standard model.

**Table 6:** Requirement of Power Distribution for FC-2 layer in VGG-16.

Operation	DRAM and CIW-ZVC modules		SRAM and processor array	
	For data movement (IFM + associate Wg)		For data computation valid IFM and Wg	
	With zero-skipping (Using MSB bit of IFM)	Without zero-skipping	With Zero-gating (Using MSB bit of Wg)	Without zero-gating
Static power (mW)	84.775	82.942	0.841	0.860
Dynamic power (mW)	21.748	113.190	16.003	19.356
Total power	106.5	196	16.84	20.21

### Design optimization and data scheduling for processor array

In the previous chapters, the data required for the processing are transferred from off-chip DRAM to on-chip SRAM through the CIW-ZVS controller. In this section, the design optimization of the processing array with 256 convolutional operators leads to improve performance (op/s), energy efficiency (ops/W), and CO utilization. The zero-valued IFMs are entirely skipped using CIW-ZVC as discussed earlier, but still, there are ~ 20% of zero values present in the weights randomly as explained in Section III. Therefore, on-chip memory contains only the valid IFMs and corresponding Wg. Some of the existing works adopted the gating method on IFM or Wg^25,26^ using a flag bit. It concentrates only on zero-valued gating to eliminate the switching activity inside the combinational modules. Due to this, energy efficiency can be improved through zero-valued gating without complex controllers.

**Fig. 16:**
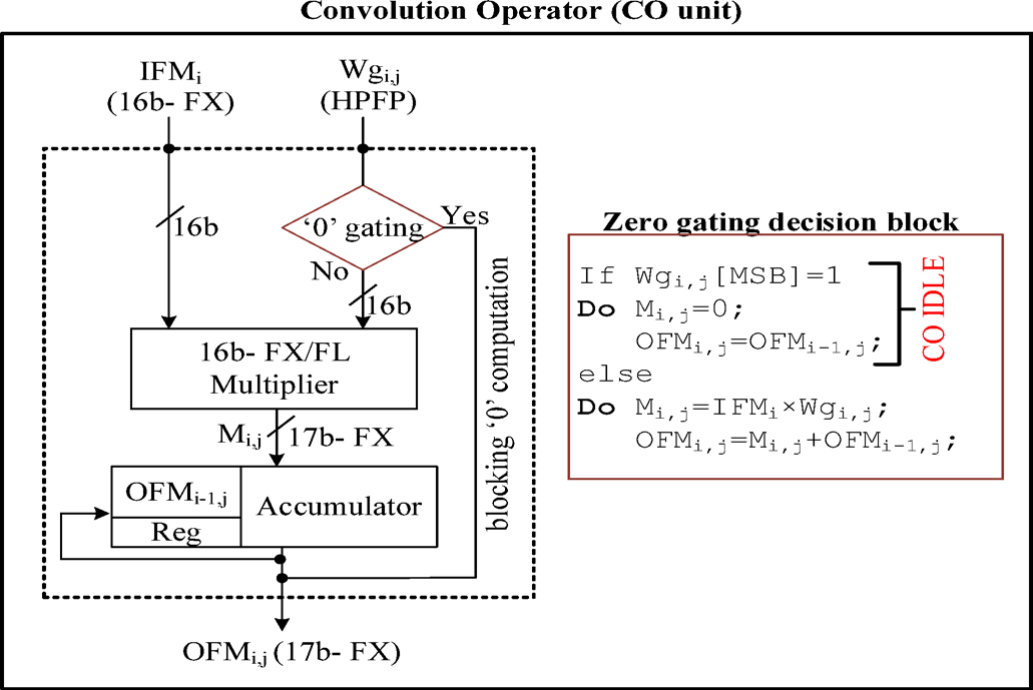
CO unit with Wg-based zero gating.

From the detailed analysis of existing works^25,26^, gating techniques is the best to implement in on-chip processor when the presence of zero values in the IFM/Wg is less than 30%. Thus, these designs focus on the trade-off between CO-IDLE status and overall energy efficiency. Consequently, the proposed design is set to process only valid IFMs and adopts the standard gating only on the Wg. Due to this, the 16-bit IFMs and 17-bit Wg are supplied for the processor array. In each CO unit, a 17th bit (MSB) of the Wg is considered a flag bit. If a flag bit is 1, the inputs (IFM and Wg) to the CO unit are gated. Else, the CO unit processes the inputs to the multiplier unit and is followed by the accumulator as shown in Fig. 16. The data-flow scheduling between SRAM and processor array is discussed in Section IV. C. Tile-based scheduling and the processing schedule of a tile of 256 IFMs and 256 × 256 Wg are illustrated in Figs. 13 and 14 respectively.

**Fig. 17:**
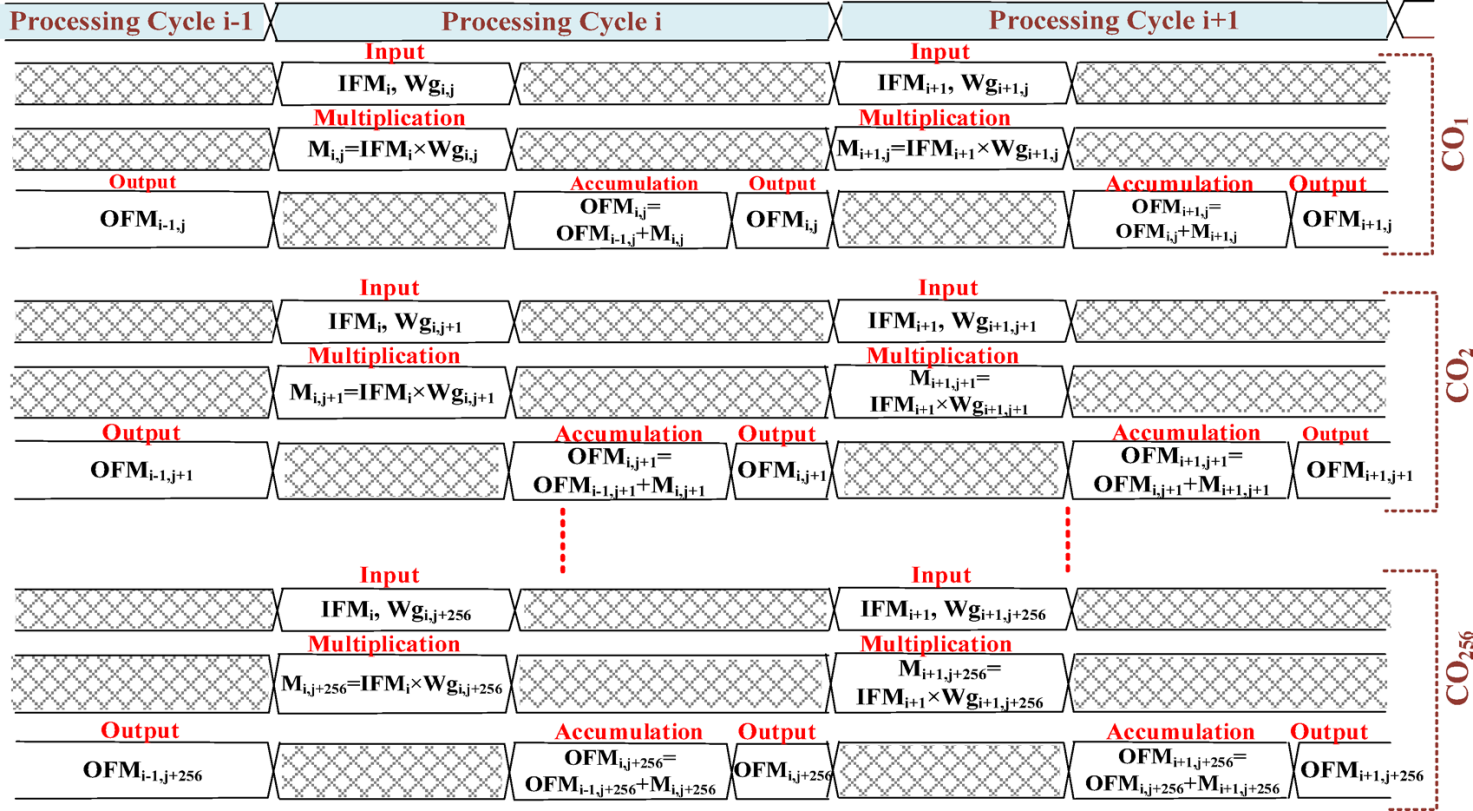
Data flow in consecutive process cycles (PC_i_, PC_i+1_).

**Fig. 18:**
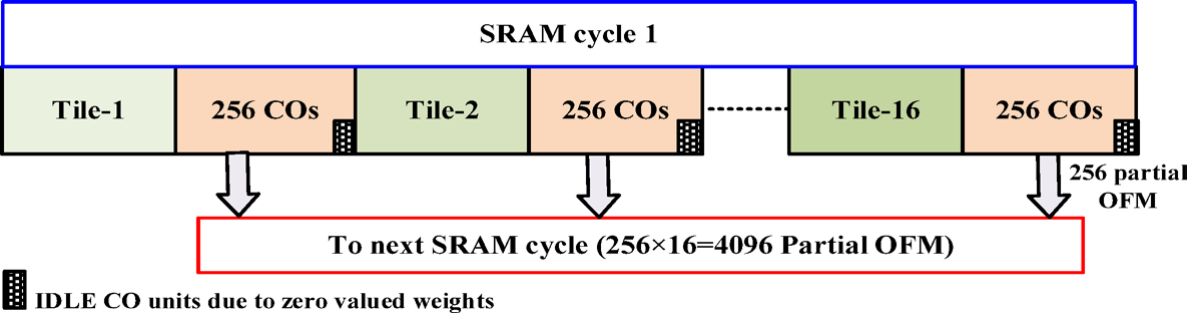
Allocation of COs for tiles 1 to 16 in an SRAM cycle.

**Fig. 19:**
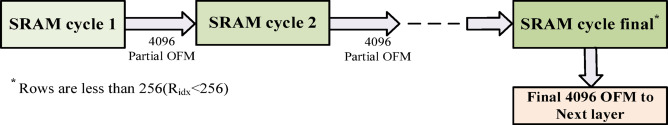
Data flow – processing an FC layer of VGG-16 with compressed data.

Here, IFM is stationary for the whole SRAM cycle (processing of tiles 1 to 16). Whenever the zero-valued Wg occurs, the gating process puts the CO unit into an idle state as shown in Fig. 16. The processor array accepts 256 IFMs and 256 Wg (one row) concurrently to 256 COs. In each CO, an *IFM*_*i*_ and a particular *Wg*_*i, j*_ are multiplied to find *M*_*i, j*_, finally accumulates *M*_*i, j*_ with *OFM*_*i−1,j*,_ and stores the partial OFM_i, j_ in the OFM register. Both multiplication and accumulation are completed in a processor cycle (PC) as shown in Fig. 17. Similarly, the same 256 IFMs and one of the columns from the 256 × 256 Wg tile are supplied to 256 COs to get processed in 256 PCs. Continuing the same process 16 times on different Wg tiles to complete the SRAM cycle is illustrated in Fig. 18. After, each SRAM cycle, 4096 partial OFMs are calculated, available in the partial OFM buffer, and ready to get accumulated in the consecutive PCs of SRAM cycles as illustrated in Fig. 19.

All SRAM cycles with full capacity (256 rows, each row has 1IFMs and 256 weights) are processed as mentioned above. The final SRAM cycle processes with less than or equal to 256 rows and needs a special arrangement. For example, there are only 100 valid IFMs and 100 rows ready for processing. To have uniformity in CO allotment and generate necessary control signals to SRAM and DRAM, the remaining 156 rows are filled with zeros. Later, zero-gating will skip computations associated with 156 rows. After all the SRAM cycles, 4096 OFMs are going to the activation layer (ReLU function) batch-wise with 256 OFMs per batch. Then, these OFMs are available for the next FC layer. The total number of the SRAM cycle has been varying based on the sparsity level in IFMs (S_IFM_) and its proposed CIW-ZVC controller for the FC layer in the CNN models.

**Fig. 20:**
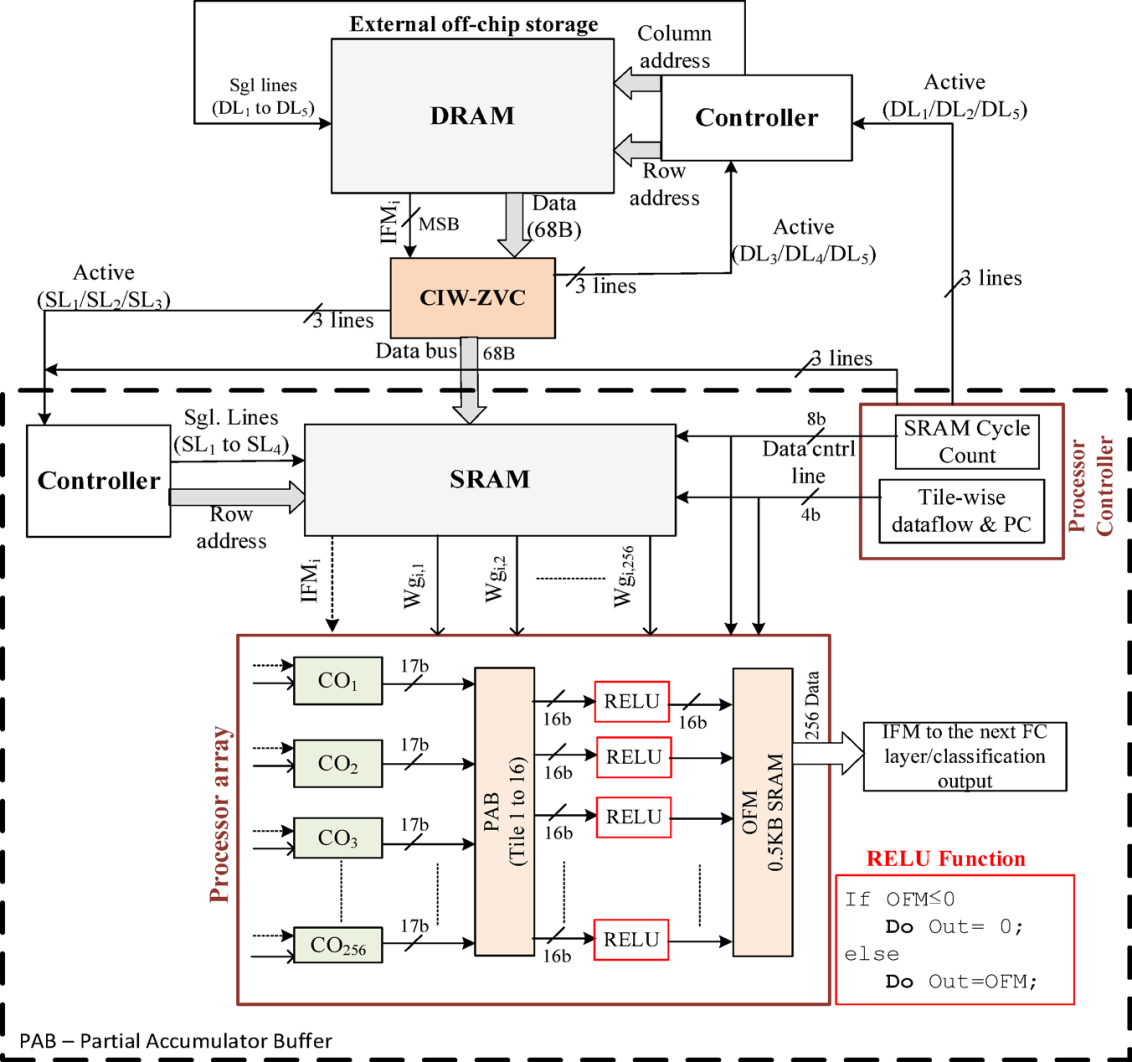
Architecture of CNN inference engine for unstructured- sparse FC layers.

### Architecture of processor array, memories, and its controllers

The overall architecture of the hardware accelerator to process the FC layer of VGG-16 is illustrated in Fig. 20. It contains the proposed CIW-ZVC controller, DRAM, and its controller in the off-chip. Also, it has SRAM, its controller, processor array, ReLU array, and OFM buffer in the on-chip. The processor array consists of 256 COs and it is scheduled continuously to process the IFMs and weights as per the strategies discussed above. After the first tile of data is processed, the processor array issues necessary control signals to the SRAM controller to start the data movement for the processing of consecutive tiles. Similarly, at the end of each SRAM cycle, the necessary control signals are issued for DRAM and SRAM as given in Fig. 12. During the final SRAM cycle, after each tile of data is processed, the final values of 256 OFMs are passed through the 256 ReLU array and stored in the OFM buffer. Correspondingly, the OFMs from all 16 tiles are passed through ReLU and stored in the OFM buffer to get processed in the next FC layers.

The proposed architecture reduces the total power consumption; improves the speed of computation, the data movement rate between DRAM to SRAM, and the energy efficiency due to the following: (1) The data transfer between the DRAM and SRAM is minimized using the proposed CIW-ZVC controller (only valid IFM and corresponding weights are moving). (2) The IFMs and weights are arranged in the SRAM by the CIW-ZVC, therefore on-chip load balancing controller is not required. (3) All valid data gets into the SRAM, and therefore it maximizes the hardware utilization of the processor array. (4) Broadcasting of IFMs among the CO units minimizes the transaction between SRAM to processor array. (5) Zero-valued weights are to be minimal to maintain the classification accuracy and managed through zero-gating. Later, it leads to a minimal number of idle states among CO units. Therefore, the hardware area is maintained to be the same.

## ASIC implementation results and discussion

The proposed architecture shown in Fig. 20 is described in Verilog HDL. Also, the stack of SRAM memories (IP in Verilog) is integrated with the processor array from the memory libraries of the Synopsys14nm technology. The simulation and functional verification of the proposed design is done with Synopsys VCS. The synthesis is done using Synopsys Design Compiler with FinFET-14 nm technology libraries and typical design effort. The power consumption is estimated with the help of the PrimeTime tool which maintains the positive slack by adding the buffer Later, the placement and routing for the proposed on-chip processor are done with IC Compiler II.

**Table 7:** Physical characteristics- our design.

**Technology**	FinFET-14nm, 125°C, RVT
Number of CO/MACs	256
Core Area (mm^2^)	0.624
Logic area (2-NAND gates)	157.7K
Frequency (MHz)	200–500
Voltage (V)	0.8
On-chip SRAMs (KB)	137
Power (mW)	7.2–16.8
Performance (GOP/s)	102.4–256

**Fig. 21:**
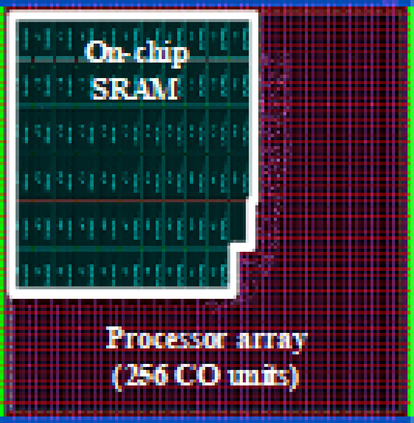
The layout of 256 MAC units.

The synthesizable clock frequency range is from 200 MHz to 500 MHz after placement and routing. However, higher clock frequency leads to a large dynamic power consumption of 16.8mW, at the lower frequency is 7.2mW. Static power is maintained to be the same at both frequencies. But, considering the peak performance, 500 MHz provides 2.5 times higher performance than at 200 MHz. Therefore, 500 MHz has been considered as the operating frequency here for further analysis. The proposed design for processing the FC layer achieves the performance and power of 256 GOP/s and 16.8mW at 0.8 V. This design supports the FC layer and activation layer directly. Figure 21 illustrates the corresponding layout and its characteristics are given in Table 7.

### Comparison with state-of-the-art hardware accelerator implementation

The performance parameters of the proposed inference for the FC layer have been compared with state-of-the-art inference accelerators that support the sparsity at input data (IFM and Wg). The proposed accelerator with CIW-ZVC exploits sparsity by compressing IFMs and their associated Wg as shown in Fig. 8. Later, zero-gating is applied on data for FC layers. The design employs a 16b-FX/FL precision for data computation and 17b-FX for data storage. Table 8 provides a summary of the proposed results and compares this work with state-of-the-art processors^10–12,16,27^, and^28^. The proposed on-chip processor array has energy efficiency and area efficiency of 15.2 (TOP/W) and 410 (GOP/s/mm^2^) respectively when the performance is 256 (GOP/s).

**Table 8:** Comparison with existing CNN accelerators

Characteristics	[10]	[11]	[12]	[15]	[16]	[27]	[28]	Proposed
Zero-value skipping/gating method	Skipping: IFM Gating: -	Skipping: IFM + Wg Gating: -	Skipping: - Gating: IFM	Skipping: IFM + Wg Gating: IFM + Wg	Skipping: Wg Gating: -	Skipping: IFM Gating: -	Skipping: IFM Gating: Wg	Skipping: IFM + Wg Gating: Wg
Sparsity handling method	SM with NZVL	AIM Coarse-grained	-	Two-symbol Huffman	RLE, COO	SM with NZVL	Tile-based compression	CIW-ZVC
Technology	28 nm	16 nm	65 nm	40 nm	65 nm	40 nm	40 nm	14 nm
Core size (mm^2^)	6.3	2.3	2.024	1.44	7.8	9	2.044	0.624
	1.575^a^	1.760^a^	0.506^a^	0.243^a^	0.361^a^	1.1^a^	0.250^a^	0.624^a^
Operating freq. (MHz)	500	260	200	200	100	320	2	500
Voltage (V_dd_)	1.0	0.8	0.8	0.9	0.9	0.9	1.1	0.8
Data type (bits)	16-FX	16-FX	8-FX	16-FX	8-FX	12-FX	16-FX	16-FX/FL
Power (mW) ^b^	155	16.3	72	121	99	151.2	0.708	16.84
	49.6^c^	14.2^c^	36^c^	33.46^c^	16.8^c^	41.8^c^	0.131^c^	16.84^c^
SRAM (KB)	1088	280.6	-	118	196	339.5	48	137
Number of CO/MAC	128	252	-	256	64	324	48	256
Performance (GOP/s)	128	131.25	90	102.4	12.8	207.3	0.192	256
Energy efficiency, (TOP/W)	0.825	8.05	1.25	0.846	0.129	1.37	0.27	15.20
	2.57^d^	9.19^d^	2.5^d^	3.06^d^	0.757^d^	4.95^d^	1.458^d^	15.20^d^
Area efficiency (GOP/s/mm^2^)^e^	81.26	74.57	177.8	421	53.45	188.4	0.76	410

^a^ Scaled based on 14 nm,$\:cor{e}^{{\prime\:}}=core\times\:{T}^{{\prime\:}}$. Where,$\:{T}^{{\prime\:}}={\left(\frac{{Tech}_{new}}{{Tech}_{old}}\right)}^{2}$[31]; 2-input NAND gate: 1GE$\:\left({0.3105\:\mu\:m}^{2}\right)$for FinFET 14 nm Technology.

^b^ Power including SRAM; ^c^ Scaled, using the model$\:{P}^{{\prime\:}}=P\times\:\left[{Tech}_{new}/{Tech}_{old}\right]\times\:{\left[{V}_{dd,\:\:new}/{V}_{dd,\:old}\right]}^{2}$^29^. .

^d^ Normalized energy efficiency based on the 14 nm scaling technique$\:{EE}^{{\prime\:}}=EE\times\:\left[{Tech}_{old}/{Tech}_{new}\right]\times\:{\left[{V}_{dd,\:\:old}/{V}_{dd,\:new}\right]}^{2}$has adopted from^30^.

^e^ Calculated the area efficiency using scaled values; GOP/s- Giga Operation per Second, TOP/W- Tera Operation per Second Per Watt.

The hardware area of the on-chip processor depends on SRAM size, data format, the number of CO units & its controller. Compared to^10,11,27^, the core area of the proposed design improves from 1.7 times to 2.8 times. The existing design^11^ uses associative index matching (AIM) address encoding and sequence decoding units to match the valid IFM and Wg pairs before processing it to the processor array and for parallel computation. Also, 252 COs are arranged in four processor cores to provide compatibility for the both convolutional and FC layers. The SRAM size in the^11^ is twice that of the proposed design. Likewise, in^10^, the capacity of the SRAM is 7.9 times higher than the proposed design. Additionally, the design needs 32-bit precision during the intermediate computation in the processor core^27^. adopts a sparsity map with a nonzero value list (SM-NZVL) for the index matching and load balancing on the IFM sparsity in the convolutional and FC layer in VGG-16. It needs additional SRAM for sparsity mapping and partial sums. Thus^27^, desires an extra SRAM capacity of 2.4 times that of the proposed design and uses the 12b of cascade precision with zero skipping based on approximate computation. Compared to the proposed accelerator, the design degrades less than 2 times accuracy for the VGG-16 model using the ImageNet dataset.

1.7 times to 2.5 times of area overhead occurs in the proposed design compared to^15,16^, and^28^. This is due to the lesser CO units and SRAM capacity in the on-chip core^16,28^. designed with 5.3 times and 4 times lesser CO units. Also, the design of^16^ adopts only the 8-bit FX. The requirement of SRAM in^15,28^ is less than 1.16 times to 2.8 times. This area overhead in the proposed design is compromised in the area efficiency by targeting better performance.

The performance (Op./s) improvement of the design is based on the trade-off between the operating frequency and the number of CO units & their operation. The proposed on-chip processor of 256 CO at 500 MHz increases the performance from 1.9 times to 2.8 times compared to^11,15,27^ and is adopted also for the FC layers. These designs operate under less than 320 MHz to the number of CO units of more than 252 in parallel. For area efficiency, the proposed design has upgraded from 2.1 times to 7.7 times compared to the state-of-the-art of existing processors except^28^. The design of^28^ is particularly for 1D applications. The area efficiency has been formulated based on the performance of the processor and core area as estimated earlier.

The major factors for the power consumption of the processors are operating frequency, processor controller for data sparsity, and SRAM data access for processor computation. The power consumption of the proposed design reduces from a minimum of 1.9 times to a maximum of 2.9 times compared to existing designs^10,12,15,27^. The SRAM access for these processors is more than from 280KB to 1088KB for the frequency between 200 MHz to 320 MHz which is greater than 2 times the proposed design. Also, these existing processors have adopted the data sparsity of S_IFM_ and S_Wg_ in the CNN layers. It is handled using the load balancing and indexing controller of SM with NZVL^10,27^, and complex encoder and decoder models to activate the specific layer in the hybrid CNN model^15^. Therefore, these existing models consume extra power and data buffers for processing the data at each stage.

The processor energy efficiency (Ops/W) has been estimated from the performance and power consumption of the processor. The energy- efficiency of the proposed processor improves from 1.6 times (minimum) to 10.2 times (maximum) compared to the start-of-the-art sparsity-based processor as shown in Table VIII. For a fair comparison, the above discussion of the proposed accelerator with the existing seven state-of-the-art sparsity-based accelerator outcomes such as area, power, energy & area efficiency is scaled under 14 nm technology and memory libraries at 0.8 V.

**Fig. 22:**
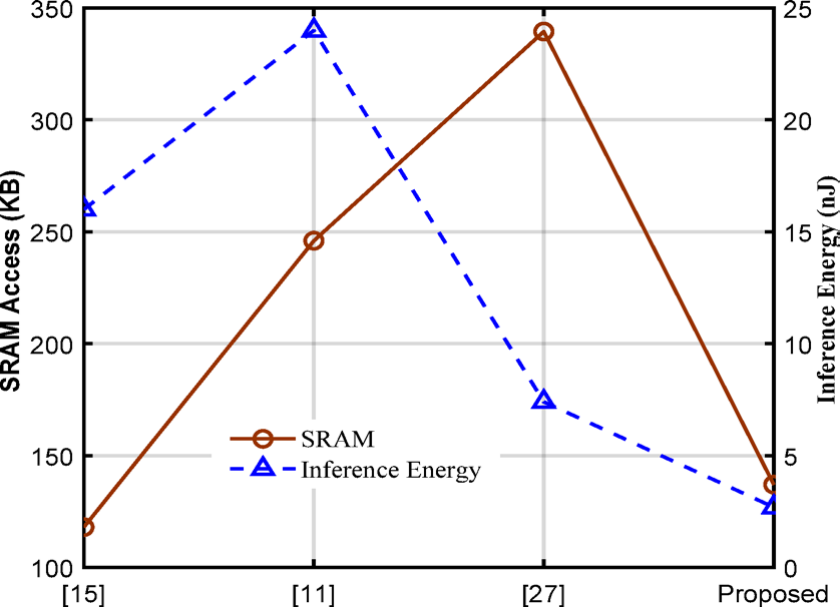
SRAM data access and Inference energy per layer for different processors.

The comparisons of implementations in the above paragraphs are with respect to the processor array only. To have a meaningful comparison the overall inference energy per FC layer has been calculated from (1) Total power consumption in mW, (2) the number of SRAM cycles, and (3) the time taken to complete one SRAM cycle. The SRAM data access and inference energy are plotted for the designs presented in^11,15,27^ and the proposed design in Fig. 22. From this, it is clear that the proposed design consumes 2.7 to 8 times less inference energy per FC layer in standard CNN model.

## Conclusion

This paper presents a dedicated hardware accelerator to process the sparse (IFM and weight) based FC layers of the CNN models. The proposed hardware has been focused on improving energy efficiency, reducing on-chip memory requirements, and optimizing data movement between the processor and memory. The analysis on an FC layer of VGG-16, with 4096 IFMs reveals that nearly 30% of its IFMs are valid (non-zero) when the validation is carried out with an ImageNet dataset. Power consumption could be further improved by adopting a MATLAB-based induced-sparsity-weights (IS-Wg) mechanism. This method analyzed and fixed the zero sparse level (η = 10) and at the same time, the classification accuracy of 95% and F1-score of 0.96 was maintained, which are nearly equal to the baseline model of the VGG-16. Also, it resulted in nearly 20% of the weights being set to zero. A memory controller unit with the proposed CIW-ZVC accomplished compress and skip the zero-valued IFMs and their associated weights. This optimized the data movement rate between the off-chip DRAM and on-chip SRAM by 3.3 times per layer in the case of VGG-16. Due to the limited SRAM access, the CIW-ZVC transferred valid data batch-wise from DRAM. The final batch size may be less than or equal to the full SRAM size, and it is managed by the CIW-ZVC and memory controller. The data arrangements in both of the memories, Tile-based computation with IFM stationary parallel processing avoided the complex on-chip load-balancing controller. Therefore, all 256 COs are always engaged, providing 100% hardware utilization. The absence of the complex on-chip controller, weight-based zero-gated, and 16-bit FX/FP COs reduced the overall power consumption. Hence the performance is 6 times more, which leads to energy efficiency 6 times and area efficiency 7 times compared to the existing designs. To the best of our knowledge from existing works, Using CIW-ZVC, the targeted on-chip processor has not adopted any row/column indexing and load balancing for the unstructured-based sparsity- FC layers of the standard CNN models. Further, the proposed memory control and data transfer techniques can be adopted for the convolutional layers with weight parameter size of 1 × 1, which are present in ResNets and MobileNet. Also, for specific non critical applications, zero-valued weights can be introduced by threshold pruning algorithm and this could skip the corresponding IFM frames.

## Data Availability

Data will be made available on request.
